# Very-Heavy Precipitation in the Greater New York City Region and Widespread Drought Alleviation Tied to Western US Agriculture

**DOI:** 10.1371/journal.pone.0144416

**Published:** 2015-12-07

**Authors:** Travis D. Andrews, Benjamin S. Felzer

**Affiliations:** Earth & Environmental Sciences, Lehigh University, Bethlehem, Pennsylvania, United States of America; University of Vigo, SPAIN

## Abstract

Observed intensification of precipitation extremes, responsible for extensive societal impacts, are widely attributed to anthropogenic sources, which may include indirect effects of agricultural irrigation. However quantifying the effects of irrigation on far-downstream climate remains a challenge. We use three paired Community Earth System Model simulations to assess mechanisms of irrigation-induced precipitation trends and extremes in the conterminous US and the effect on the terrestrial carbon sink. Results suggest precipitation enhancement in the central US reduced drought conditions and increased regional carbon uptake, while further downstream, the heaviest precipitation events were more frequent and intense. Specifically, moisture advection from irrigation in the western U.S. and recycling of enhanced local convective precipitation produced very-heavy storm events that were 11% more intense and occurred 23% more frequently in the densely populated greater New York City region.

## Main Text

Precipitation extremes are deadly, damaging to infrastructure and ecosystems, and have prompted widespread interest in identifying anthropogenic drivers, including greenhouse gas emissions and the role of agricultural irrigation. Recent studies suggest irrigation may enhance regional precipitation [1–7], although far-downstream effects remain uncertain. Extreme precipitation events in New York City and the northeastern US have caused catastrophic flooding and impacted the millions who live in the region [8,9]. From 1958–2012 very-heavy precipitation events in the northeastern US region increased by 58% [10], and extreme precipitation is projected to further increase by 8–9% by mid-century [8]. In contrast, drought is projected to increase in the central US under all future emissions scenarios [11]. Drought reduces carbon uptake by vegetation and represents a positive feedback for anthropogenic climate change [11]. Irrigation improves agricultural resilience to drought, and it is critical to food production in arid and semi-arid regions. Evapotranspiration from irrigation represents a driver of, and response to, anthropogenic climate change that is expected to expand spatially and increase intensity in some regions [12,13]. Determining connections among commercially important activities like agricultural irrigation, with climate change and ecosystem services can inform policy and natural-resource management aimed at sustainability.

Agriculture in many regions is dependent on tremendous amounts of irrigation with implications for the global-water cycle. In 2005, agriculture in the United States resulted in the irrigation of 24 million hectares (roughly the area of the Great Lakes) with 182 km^3^ of water, an average application rate of 0.7 m Ha^-1^ year^-1^, of which, roughly half is lost to the atmosphere through evapotranspiration [14–16]. The amount of area irrigated in North America has increased somewhat exponentially over the last 150-years, expanding from approximately 1.5 million hectares in 1888 to approximately 20 million hectares in 1984 [17,18]. Similar trends also occurred in Mexico and Canada, where the irrigated area in 1984 was approximately 5 million hectares and 300,000 hectares, respectively [17]. Since the 80’s the expansion of irrigated land has generally slowed, due in part to groundwater constraints. The Ogallala aquifer, a major source of irrigation water, has decreased by about 333 km^3^ (8.5%) between 1950 and 2007 [18,19]. Projections indicate agricultural production may continue to grow using engineered water-efficient crops until 2040, at which point groundwater limitations will become severe [20].

Recent modeling studies suggest that intensive regional irrigation can influence surface air temperature, precipitation climatology, and monsoon intensity [1–4,21–23]. For example, Lo et al. (2013) found that water vapor introduced by irrigation in California's central valley is transported by wind, resulting in a 15% enhancement of monsoon precipitation in the southwestern US that could contribute up to 30% of Colorado River summer streamflow. However, Sorooshian et al. (2011) suggested that although irrigation from this region increased relative humidity 9–20%, it had little influence beyond the irrigated area. These previous model-based studies of irrigation relied on a single experiment rather than an ensemble of experiments useful to assess variability [24]. Resolving mechanisms by which irrigation can influence downstream precipitation is critical to fully understand observations and plan mitigation strategies. Generally, global and large-scale regional modeling studies suggest summer precipitation in the central US has increased due to irrigation [1–4]. The primary mechanism for this precipitation enhancement was thought to be enhanced convective precipitation as a result of increased convective available potential energy (CAPE) of the atmosphere from moister surface conditions [4,18]. At the site of irrigation, surface cooling associated with increased latent heat flux has a stabilizing effect on convective systems, which may direct convective precipitation downstream where surface temperatures are not cooled [4,18]. However, in semi-arid regions summer convective fronts can be highly unstable, in which case additional surface moisture will increase CAPE even with cooling [4]. While convective precipitation directly increases, the fate of old convective cells [25] and moisture advection into stratiform cloud systems has not been examined.

Non-convective precipitation can occur when warm moist fronts override cooler air masses to produce stratiform conditions. As the warm air mass rises, the air gradually cools and forms grey featureless clouds that lead to precipitation events ranging from mist to extreme extended events [26]. Across the US, stratiform precipitation occurs least frequently in the desert southwest and becomes more frequent approaching the northeastern US [27]. Stratiform precipitation is associated with large-scale precipitation in mid-resolution atmospheric general circulation models [26,27]. Due to the large size and energy of these systems, they have potential to move moisture long distances and deposit large amounts of precipitation [27]. Globally, the location, timing, quantity of water vapor emitted, and atmospheric residence time are largely known for agricultural irrigation; however, the pathway from irrigation to precipitation and the magnitude and the likelihood of changes associated with climate extremes have not been fully assessed in far-downstream regions. Identifying a relationship between central and western US irrigation and enhanced stratiform precipitation events in the northeastern US may inform understanding of causes contributing to observed extremes.

Here we quantified the explicit influence of irrigation on changing precipitation regimes and excluded other possible anthropogenic influences, particularly the direct effects of climate change due to elevated CO_2_. Specifically, we synthesized an ensemble of three paired Community Earth System Model (CESM) experiments and quantified the influence in the U.S. of irrigation on total precipitation, changes in terrestrial carbon uptake to understand interactions with anthropogenic emissions of CO_2_, and far-downstream influence on precipitation frequency and intensity within the northeastern U.S. We compared the results to observed trends to provide a new perspective on future water resources in the Great Plains and greater New York City regions. Modeled irrigation in the U.S. was most intense during the summer months of July and August and thus all results were for these months (hereafter referred to as peak-summer).

## Models and Methods

We performed three experiments with paired global simulations using the National Center for Atmospheric Research Community Earth System Model (NCAR-CESM). CESM is a state-of-the-art fully-coupled global model of the land, atmosphere, ocean, and sea ice (further described by Hurrell et al. 2013) [28]. The Community Land Model version 4.0 (CLM) was extended to incorporate a carbon-nitrogen (CN) biogeochemical model that considers the effects of nitrogen limitation on terrestrial carbon cycles [29,30]. The recently updated CLM version 4.5 was not available when the computationally-intensive spin-up of the CN model (required for irrigation effects in CLM4.5) was started. Improvements in nitrogen dynamics in CLM4.5 may reduce ecosystem transpiration and the magnitude of downstream effects, similar to the effects found in experiments using CLM4.0 without the CN model (discussed below). We coupled CLM4 to the Community Atmosphere Model versions 4.0 (CAM4) and 5.0 (CAM5) [31], prescribed sea-surface temperatures, and prescribed sea-ice (experimental design described below).

Prescribed model components are based on the Atmosphere Model Intercomparison Project [31], rather than prognostic model computations. CLM version 4.0 incorporates an interactive irrigation model that dynamically responds to climate [32]. With irrigation activated, cropland area of each grid cell is divided into an irrigated and unirrigated fraction based on the availability of irrigation in the area [33], further described in Levis and Sacks (2011). The model checks if water is limiting for photosynthesis the first time step after 6 AM local time and calculates target soil moisture. This amount of water is applied at a constant rate to the ground surface over the following four hours. Ground application was chosen because it mimics flood irrigation, the most common form of irrigation globally [22]. In 2005 the US used of mix of irrigation types including flood irrigation on ~10.7 million hectares, sprinkler irrigation on ~12.3 million hectares and micro-irrigation on 1.6 million hectares [15]. Sacks et al. (2009) experimented adding irrigation as precipitation with canopy interception and found the model “relatively insensitive,” possibly because ground evaporation was determined to be unexpectedly high. Water for irrigation is removed from nearby rivers. Annual irrigation amounts are validated with observed gross irrigation water use around the year 2000 (2500–3000 km^3^ year^-1^ [34]).

**Fig 1:**
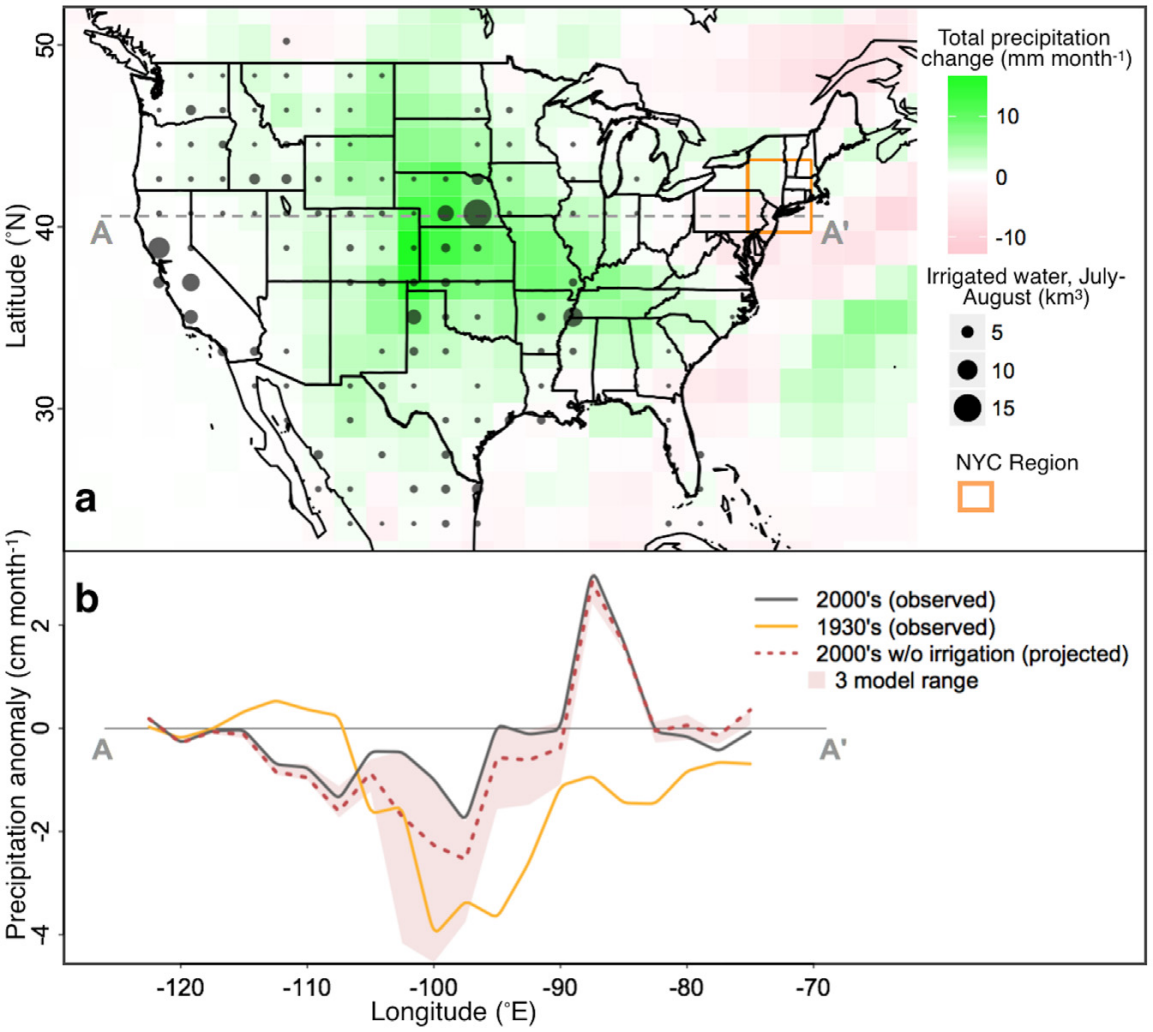
Three-experiment mean difference between paired irrigation and control simulations in peak-summer (July-August) total precipitation (a). The amount of irrigated water applied within the grid-cell is shown with grey circle size. The A-A’ transect shows the location of the precipitation anomalies investigated in (b). Inset b shows observed US mean peak-summer precipitation anomalies for an east-west transect at 40.5°N latitude, compared to 1901–1930 climatological mean, for the decades of 2000–2010 (solid grey line) and 1930–1940 (solid orange line). Projected 2000's precipitation anomalies without the influence of irrigation (dotted brown line; 3-model mean) are shown with the 3-model range (shaded area). The wide 3-model range indicates irrigation effects on convective precipitation were highly sensitive to the different experimental conditions, which may represent a possible range of natural variability and/or uncertainty in irrigation modeling studies.

All experiments used the same 1.9 x 2.5 degree resolution to minimize complexity and computational resources yet allow for downstream effects and feedbacks between the land and atmosphere. We found the moderately coarse resolution was sufficient to identify effects of large-scale processes (i.e. irrigation) across continental spatial scales. Limitations of coarse resolution, including underestimates of extreme precipitation [35] were avoided in analyses of the greater New York City region where results were primarily based on relative change of large-scale stratiform precipitation rather than small convective storms. The greater New York City region was defined as the 4-grid cell area encompassing Northern New Jersey, New York City/Long Island, Upstate New York, Lehigh Valley, Pennsylvania and adjacent areas (NYC region; see Fig 1). The model resolution allowed quantification of precipitation changes within the NYC region, but not exclusive to the City of New York. High resolution (i.e. resolution < 25 km^2^) global experiments would have been computationally costly, and precipitation biases in CESM can worsen at increased resolution [36]. Recent irrigation studies that used high-resolution regional climate or weather models [3–6,37], identified more precise intra-regional effects using lateral boundary conditions that precluded continental scale analyses [16].

Model experiments compare CESM results for scenarios with and without global irrigation (herein referred to as control and irrigation simulations, respectively) for the year 2000 using three different CESM versions and scenarios so that the effects of irrigation and natural variability on precipitation climatology and other earth-system characteristics can be partitioned and compared. The experimental design was based on simple and conservative replication. Instead of replicating the same (non-deterministic) paired experiment three times; we ran three different paired experiments that should produce near-replicate results using slightly different parameters. The intention is not to compare the effects of model parameters, but rather use the range of results as a conservative estimate of replication. Each experiment was run for 100 years and climatological averages were calculated for peak-summer (July-August). These averages were then used as the basis for comparison between experiments. Early exploratory studies of total precipitation changes in June and September suggested weakly defined differences, whereas winter months showed minimal difference. Experiments are summarized in Table 1 and as follows:

**Table 1:** Experimental setup summary.

Experiment Number	Simulation	Carbon/Nitrogen Coupling?	Simulated Irrigation	Temporal Data Output	Model Version
1	Irrigation	Yes	Global	Monthly	CAM4/CLM4CN
1	Control	Yes	None	Monthly	CAM4/CLM4CN
2	Irrigation	No	Global	Monthly	CAM5/CLM4
2	Control	No	None	Monthly	CAM5/CLM4
3	Irrigation	No	Western N. America	Daily	CAM5/CLM4
3	Control	No	None	Daily	CAM5/CLM4

Experiment-1: In the first experiment, CAM4 and CLM4 were coupled to the CN model with fully prognostic ecosystem feedbacks and quantified change in carbon and nitrogen sinks and fluxes. The CN model has known model biases including the potential for overestimated transpiration [30]. The experimental design minimizes the effect of CN model biases by comparing paired experiments and by averaging results with two experiments that excluded CN coupling (i.e. Fig 1). The use of irrigation with CLM-CN has not been described before in the literature, nor has it been validated by NCAR. Therefore an equilibration spin-up (~1600 model years) and validation with global irrigation values was required before evaluating model outputs. Equilibrium was determined once global net ecosystem productivity (NEP) was not trending in any direction and was near zero, indicating the carbon and nitrogen cycles were equilibrated, at which point, the models were run for an additional 100 years to calculate climatological averages. The model spin-up used year 2000 land use conditions. Global data output was monthly.

Experiment-2: The second experiment attempted to replicate the findings from Experiment 1 using the newer CAM5 coupled to CLM4 without the CN model. Global data output was monthly. This experiment excluded the carbon-nitrogen model coupling and was spun up for 10-years to allow control and irrigation runs to differentiate, then run for 100 years to calculate climatological averages, following methods adapted from Lo et al. (2013).

Experiment-3: The third experiment attempted to replicate findings from the previous two experiments using the CAM5 coupled to CLM4 with irrigation limited to the western US (rather than global) for more detailed investigation into the timing and downstream reach of irrigation from the western US. Irrigated areas were between -125W and -95W longitude or roughly from California to eastern Kansas, including small areas of irrigation in southern Canada and northern Mexico. This experiment had both monthly and daily global data output, Following a 10-year spin up, the experiment was run for 100 years to calculate monthly climatological averages as well as to identify daily precipitation extremes from the daily output data.

All statistical analyses were performed using R version 3.1.2 [38]. We calculated the mean differences between paired simulations to identify the influence of irrigation and then determined significant correspondence between mean effects in the three experiments with a t-test (n = 3). From the 100-year July and August daily data (Experiment 3), we categorized large-scale, convective, and total precipitation intensity into none-median, median-heavy, heavy, and very heavy using 0–0.5, 0.5–0.99, 0.99–0.995 and 0.995+ quantiles of 1-day precipitation quantity, respectively. Change in storm frequency was calculated as the difference between total number of events in the irrigation and control simulations that occurred within intensity categories (i.e. a range of storm precipitation quantities) defined by the control simulation. Storm frequency standard error was calculated by bootstrap sampling the full datasets and recalculating change (1000 iterations). Storminess was quantified from the daily data using the bandpass filtered (2–6 day) standard deviation of the atmospheric 500 mb pressure height for all days and for days with very-heavy stratiform precipitation. Individual storm tracks were manually identified from the daily data output of the irrigation simulation (Experiment 3) using low atmospheric surface pressure and/or precipitation, tracked four days before and one day after very-heavy precipitation events that occurred within the 4-grid cell area encompassing the NYC region.

The spatial distribution and magnitude of modeled total precipitation changes were put in context using an east-west transect across the US centered on 40.5°N latitude (± 0.75° latitude) that compared total precipitation in July and August for the modern period (2000’s), a hypothetical modern period without irrigation-enhanced precipitation, and the Dust Bowl decade (1930’s) to the period from 1901–1930. Observations were based on University of Delaware compiled and modeled historic monthly precipitation 0.5°x0.5° gridded data (esrl.noaa.gov/psd), spatially averaged to match the CESM model resolution. Decadal means for the 1930’s and 2000’s were compared to mean precipitation from 1901–1930. The 1901–1930 comparison period was chosen to represent a 30-year climatological mean with minimal influence from irrigation. The 1930’s decade was an exceptionally dry period in US history and was used for comparison. The 2000’s represent modern climate with full irrigation influence and are expected to be the closest comparable climate to the modeled irrigation simulations.

## Results and Discussion

**Fig 2:**
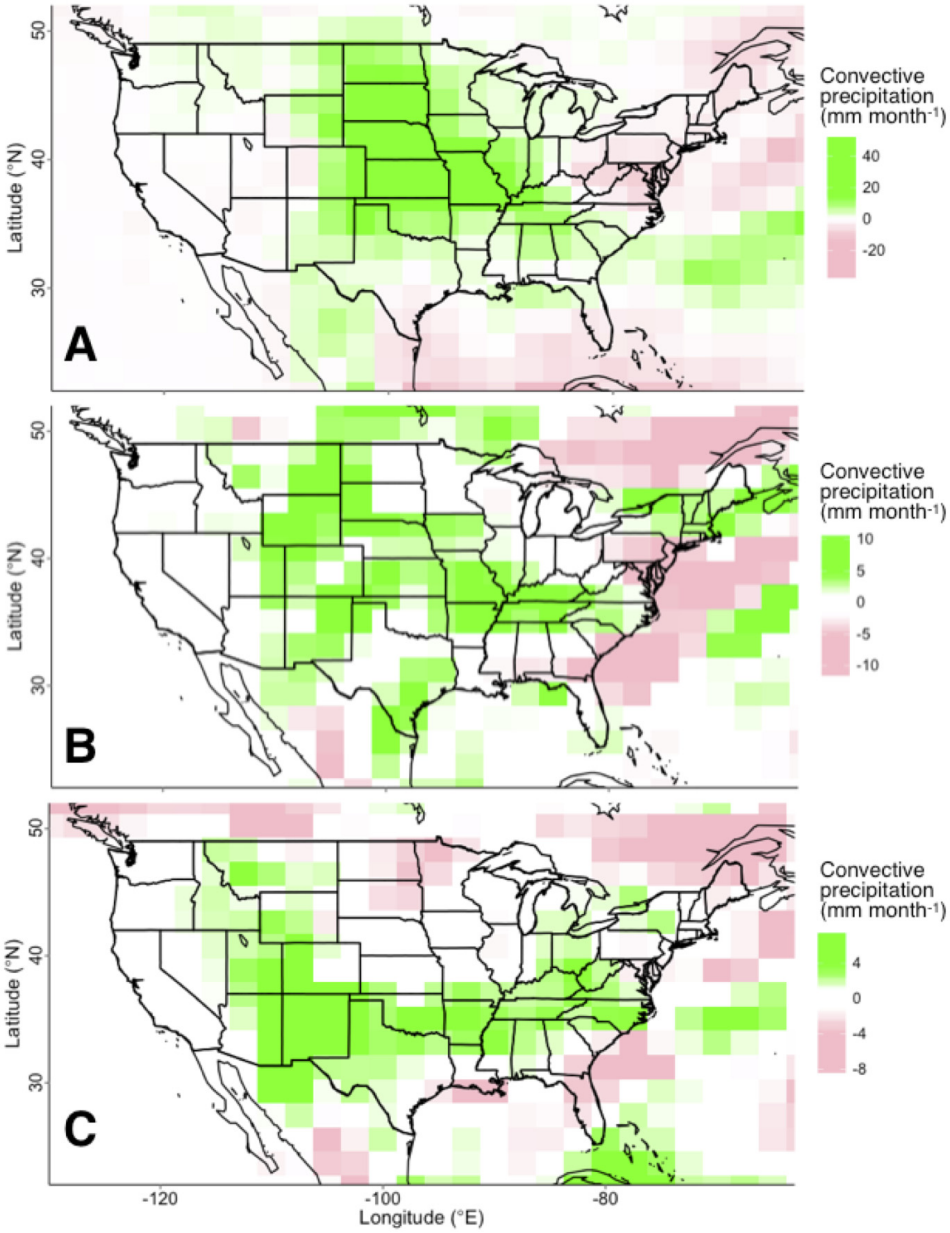
Mean difference between paired irrigation and control simulations in peak-summer (July-August) convective precipitation for (A) Experiment 1, (B) Experiment 2, and (C) Experiment 3. Irrigation consistently enhanced convective precipitation in all three experiments but the magnitude and location of enhancement were different. Since all three experiments should be reasonable measures of irrigation effects, we considered the average across experiments to be the simplest and most robust measure of average irrigation effects in the continental US (i.e. Fig 1a).

All three experiments showed July and August precipitation enhancement spanning from western to southeastern states (Fig 1). The enhancement was dominated by convective precipitation, which has been attributed to increased surface-based CAPE from irrigation-associated evapotranspiration [4,5,7,16,18]. Additional moisture in the atmosphere attributed to irrigation-enhanced evapotranspiration is hereafter referred to as iET. The amount of enhanced precipitation in the central US was equal to approximately 17% of the total amount of iET, which compares to 15.8% found by Harding and Snyder (2012) and suggests the majority of iET rains out further downstream. Interestingly, the location of greatest precipitation enhancement did not overlap well between experiments with enhanced convective precipitation focused over the central Great Plains in Experiment 1, distributed from the Rocky Mountains to Tennessee in Experiment 2 and distributed from Arizona to North Carolina in Experiment 3 (Fig 2). Experiment 1 produced the greatest precipitation enhancement in western Kansas, likely attributable to a simpler atmospheric model (CAM4) and carbon and nitrogen cycle feedbacks. Experiment 3 produced the largest quantity of added precipitation in the northwestern Texas panhandle, similar to Lo et al. (2013), that attributed this large precipitation anomaly to moisture advection from irrigation in the Central Valley of California. Experiments 2 & 3 had the largest difference in the location of enhanced precipitation. We interpret this to indicate western US iET primarily enhanced existing convective systems, as suggested by Huber et al. (2014), and the location of these convective storms, were influenced by global irrigation through slight changes in global circulation patterns. Overall, we expect iET is likely to rain out across a large area of the central US and therefore the average of the three experiments should best capture this distribution (Fig 1).

We projected how summer precipitation would be different if irrigation continued at early 20^th^ century levels in the western US based on a simple assumption that regional climate would have be similar except for the addition of precipitation attributed to irrigation. This projection can provide a reference for water resources management if future irrigation intensity were reduced. Peak-summer precipitation anomalies (divergence from 1900–1930 mean precipitation) suggest the central US has had slightly below-average precipitation recently. By subtracting the amount of modeled precipitation due to irrigation from the observed means, drying in the Great Plains region ranged from unchanged to drier than extreme dryness of the 1930’s. The range of model results highlights the sensitivity of precipitation enhancement to global circulation patterns and vegetation feedbacks. On average, the central US would have been subject to a more intense drying trend without the widespread influence of irrigation. While many observed records over the past few decades are thought to represent the early onset of greenhouse gas-driven climate change [9,39], we suggest the early onset of climate change in the Great Plains has been partially masked by the effects of irrigation. A similar sentiment was described for the effect of irrigation on temperature in California, where future water scarcity could cause agricultural abandonment and warmer average temperatures [40]. In the Great Plains projected future water-resource limitation would not only directly reduce irrigated crops, but it would also have implications for regional food security and ecological sustainability non-irrigated lands.

**Fig 3:**
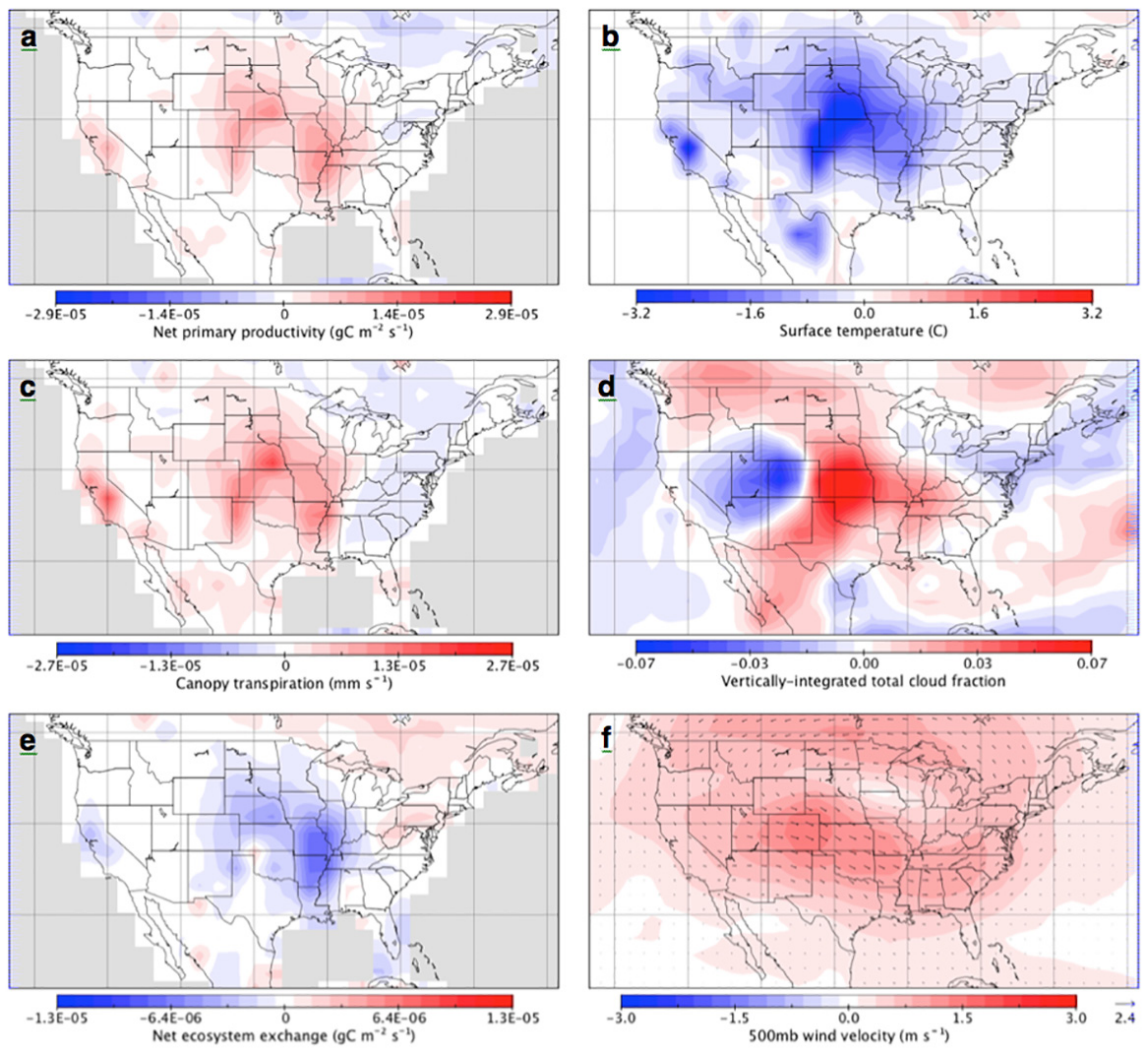
Mean difference between the paired irrigation and control simulation of Experiment 1 (CLM4 with coupled carbon and nitrogen cycles), for select ecosystem and climate parameters during peak-summer (a-f). Note, for net ecosystem exchange (e), ecosystem uptake is negative.

Reduced drought effects in the central US from enhanced convective precipitation also influenced temperature, wind and cloudiness, and resulted in substantial changes in ecosystem transpiration, net primary productivity (NPP), and net ecosystem exchange (NEE; Fig 3). NPP and NEE, which is a measure of carbon sequestration, were enhanced in the central US, consistent with semi-arid vegetation and agricultural crop response to increased moisture, despite increased cloudiness and lower temperatures that generally impede plant growth in the CLM-CN model. Irrigation had an overall cooling effect during peak-summer that extended across the surface of the entire continental US (Fig 3) and well into the atmosphere. This cooling from increased latent heat flux and increased cloud cover generally stabilizes the atmosphere and slows convection [4,16,22]. Overall, irrigation resulted in a net uptake of carbon in the central US during peak-summer (i.e. negative NEE), and a net loss of carbon from parts of the eastern US and Canada. Reduced NEE in Canada relates to reduced net primary productivity (Fig 3a) and increased microbial decomposition (not shown), likely due in part to warming. Thus the interaction of irrigation effects with climate warming and elevated CO_2_ was spatially complex.

**Fig 4:**
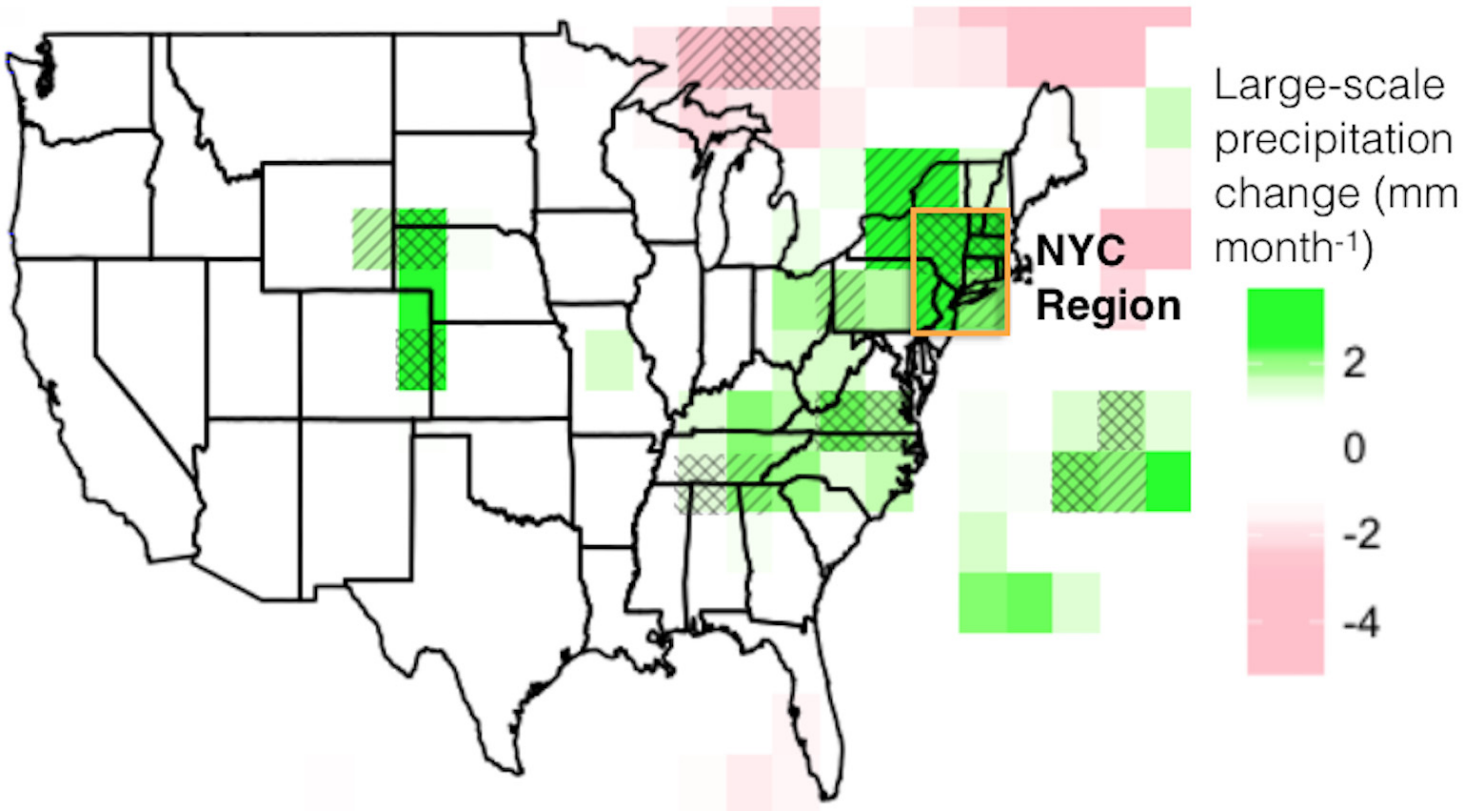
Three-experiment mean difference between paired irrigation and control simulations in peak-summer stratiform precipitation, with significance correspondence between models shown with hatching (p < 0.1) and cross-hatching (p < 0.05). The four grid cells that compose the NYC region are outlined with an orange box.

**Fig 5:**
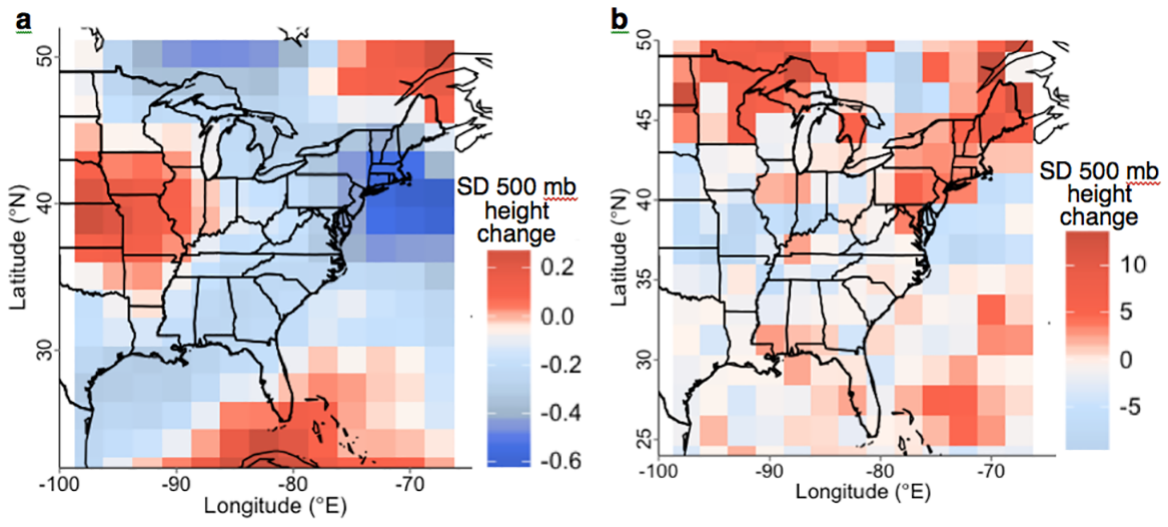
Mean difference in the 500 mb pressure height standard deviation between the paired irrigation and control simulations in peak-summer from daily data (Experiment 3) for (a) overall climate, and (b) days with very-heavy large-scale precipitation in each grid cell. Higher values indicate more frequent and/or intense storm events.

In the northeastern US total precipitation change due to western and central US irrigation was negligible overall; however, large-scale stratiform precipitation occurred more often, was more intense, and was consistently enhanced among experiments in the NYC region (Fig 4). Within this region, daily precipitation statistics suggest very-heavy stratiform systems occurred 31.0% more often and were 15.8% more intense in peak-summer (Table 2). Storms that produced the most total precipitation were generally a mix of stratiform and convective precipitation, and occurred 23.1% more often and were 11.3% more intense in the NYC region (Table 2). Overall storminess and cloudiness was reduced in the NYC region (Figs 5a and 4d, respectively) reflecting the significant reduction in frequency of median-heavy convective storms (Table 2). However, intensity and frequency of storms that produced very-heavy stratiform precipitation was higher in the northeastern US and western Great Lakes region (Fig 5b). These results correspond to the above calculations for the NYC region (Table 2) and suggest the area of increased downstream storminess extends to other regions under the conditions of Experiment 2 (limited irrigation area and daily data output).

**Table 2:** Change in storm frequency and intensity in the NYC region. Note, the none-median category includes days that had zero precipitation, while very-heavy events are the top 0.5% of daily precipitation that occurred in July and August during the 100-year simulation. Significant difference from the control simulation (p < 0.05, bold values) was determined using bootstrapped standard error (1000 iterations) for storm frequency, and a t-test for storm intensity.

		Storm frequency		Mean storm intensity		
Precipitation Type	Storm event intensity	Control storm probability	Irrigation difference (%)	Precipitation (mm day^-1^)	Irrigation difference (mm day^-1^)	Irrigation difference (%)
Stratiform	none-median	0.490	**-1.9**	0.01	+0.0003	+4.0
Stratiform	median-heavy	0.490	+0.7	0.73	+0.01	+1.0
Stratiform	heavy	0.005	-7.1	14.13	+1.59	**+11.3**
Stratiform	very-heavy	0.005	**+31.0**	25.65	+4.05	**+15.8**
Convective	none-median	0.500	**+1.4**	0.12	-0.01	**-12.3**
Convective	median-heavy	0.490	**-1.6**	3.77	-0.01	-0.1
Convective	heavy	0.005	+19.0	14.57	+0.24	**+1.6**
Convective	very-heavy	0.005	+0.9	18.06	+0.24	+1.3
Total Precipitation	none-median	0.500	+0.8	0.11	-0.01	**-6.8**
Total Precipitation	median-heavy	0.490	-1.1	4.67	+0.02	+0.4
Total Precipitation	heavy	0.005	-4.9	20.71	+1.17	**+5.6**
Total Precipitation	very-heavy	0.005	**+23.1**	30.97	+3.49	**+11.3**

Storm tracks of the 20 most intense stratiform storms in the NYC region traversed the continental US most commonly from west to east, often initially producing some degree of convective precipitation in the Great Plains region and then changing to large-scale stratiform precipitation as the storm approached the northeastern US. This storm pathway suggests a mechanism where by “vigorous” convective storms transitioned into “older” convective systems and eventually formed stratiform precipitation, as originally described by Houze (1997). Specifically, results suggest iET and regionally enhanced transpiration (due to vegetation feedbacks; Fig 3c) advection directly increased the size of convective storms over the Great Plains region, however as these storms moved downstream, iET no longer enhanced CAPE, and the energy and updrafts supporting these convective systems were reduced, resulting in a collapse of residual moisture into large stratiform systems. Similarly, convergence and convective lift of Great Plains moisture into low pressure warm fronts moving downstream and interacting with cold air masses would set up enhanced stratiform precipitation without the convective storm precursor. Large stratiform storms with unclear trajectories, or those with warm low pressure fronts that tracked through Canada or along the eastern US coast, may not have been influenced by irrigation. However, stratiform precipitation can also be enhanced when precipitation falls through cool air masses with increased moisture. Further when large-scale systems move over moist regions with high surface humidity, such as the northeastern US, enhanced condensational heating intensifies the energy in these systems [36].

## Conclusions

Agricultural irrigation is critical to maintain global food supply and knowing its influence on climate can inform preparedness in assessing water availability issues and changing ecosystem services. This work adds to mounting evidence that irrigation enhances regional precipitation and also finds far-downstream effects. In the Great Plains, additional summer precipitation may slightly reduce ground/surface water withdrawal needed for irrigation, making agriculture (up to ~8%) more sustainable. However the location of greatest total precipitation enhancement varied between experiments. Further investigation into the controls on spatial variability may inform climate prediction and preparedness for changes in global irrigation patterns. Agreement of experiments in far downstream large-scale precipitation enhancement suggests a consistent mechanism. We attribute the far-downstream effect of irrigation to have caused a substantial portion of the observed intensification of very-heavy precipitation events the NYC region [10]. As agriculture continues to intensify over then next few decades, downstream residents should prepare for more extreme precipitation and flooding. Notably, the relative influence of greenhouse gasses on NYC regional extremes is lower than previously suggested [9] and the effect of emissions-based climate-change mitigation efforts may have less of an effect than anticipated. Of the numerous positive effects of irrigation, we speculate cooling associated with iET and evaporation of enhanced regional precipitation stabilizes the central US atmosphere in mid to late summer and would make conditions for tornado formation less favorable. Questions and uncertainty remain and future work should utilize a greater number of ensemble simulations run for longer periods at variable resolution to assess controls on variability and extremes.
